# Ecstasy-Induced Rhabdomyolysis Leading to Severe Acute Kidney Injury Requiring Temporary Hemodialysis: A High Risk for Recurrence With Repeated Exposure

**DOI:** 10.7759/cureus.64010

**Published:** 2024-07-07

**Authors:** Sabastain F Forsah, Bibi Razak, Divine Besong Arrey Agbor, Gnama Kouyate, Tristan Cossaro, Sonia Dadlani, Anum Humayun

**Affiliations:** 1 Internal Medicine, Richmond University Medical Center, Staten Island, USA

**Keywords:** methylenedioxymethyl-amphetamine, drug use, hemodialysis, rhabdomyolysis, aki, ecstasy, mdma

## Abstract

Ecstasy (3,4-methylenedioxymethyl-amphetamine, MDMA) is an illicit drug that has found widespread use. It is mostly used by adolescents and young adults, particularly during intense and prolonged dance parties for its mood-enhancing properties. Despite these pleasurable effects, users may have potentially serious side effects including death. One of the serious side effects is rhabdomyolysis, which can proceed to severe acute kidney failure. Due to different personal characteristics, some individuals taking the same dose of MDMA may experience more adverse effects than others. Individuals who experience adverse effects are more likely to experience them with each use. Our patient used MDMA two times in his life, and on each occasion, he had severe rhabdomyolysis with severe acute kidney injury (AKI) requiring temporary hemodialysis. Health professionals should screen all adolescents and young adults for illicit drug use during each encounter and counsel them against it.

## Introduction

Illicit drugs are psychoactive substances banned by international drug control treaties and whose non-medical use poses unacceptable risks to the people using them. The incidence of illegal drug use is rising especially in the adolescent population. Its use peaks in the mid-adolescence and early adulthood. They are mainly used at “raves,” intense and prolonged dance parties. Drugs frequently abused are Cannabis, ecstasy, nitrous oxide, and cocaine [1].

Ecstasy (3,4-methylenedioxymethyl-amphetamine; MDMA) is an illicit synthetic drug that has found widespread use in young adult populations. MDMA is a phenethylamine, and it shares properties with both amphetamines and hallucinogenic drugs [2]. MDMA was first synthesized as an appetite suppressant in 1914 but it was never used for that purpose. Rather, it was more used as a recreational drug with its use as a recreational drug first officially noted in the 1970s. It was subsequently banned in 1985 by the US Drug Enforcement Agency. However, it is still third on the list of most-seized drugs after hashish (cannabis sativa and derivatives) and heroin [3]. MDMA has stimulant and hallucinogenic effects and it is taken for its mood-enhancing properties causing: euphoria, empathy, enhanced sociability, heightened mental awareness, feelings of vibrancy and well-being, and the loss of anxiety, emotional fluctuations, and indecisiveness [2,3]. Despite these pleasurable effects, users also have undesirable effects and can present with lack of appetite, jaw clenching, dehydration, tachycardia, drowsiness, muscular spasms, anxiety, impulsiveness, paranoia, muscle spasms, and mood changes that can lead to aggression and crime. Serious adverse effects can also occur and could potentially be life-threatening. Patients can have acute hyponatremia, fatal hyperthermia, disseminated intravascular coagulation (DIC), non-traumatic rhabdomyolysis, malignant hypertension, and even sudden death [2,3]. We present a case of a 45-year-old male who after taking MDMA presented with extremity pain. He was found to have rhabdomyolysis which progressed with severe acute kidney injury (AKI) requiring hemodialysis. This was the second occurrence, and after each exposure, the patient required hemodialysis following just a one-time consumption.

There have been many reported cases of rhabdomyolysis after MDMA use. However, cases of recurrent MDMA-induced rhabdomyolysis which led to severe AKI requiring dialysis in the same individual after each exposure are rare. This case strengthens the fact that illicit drugs can have serious and even fatal side effects and therefore screening and counseling are advised in high-risk patients with the goal of discouraging their use. This case also shows that there are some patients with increased susceptibility to the adverse effects of MDMA and they often experience more severe side effects with each exposure.

## Case presentation

A 45-year-old male presented with agitation and paranoia and later attested to ingesting MDMA about nine hours prior to coming to the emergency room. He reported an inability to move his upper and lower extremities due to muscle pain but denied loss of consciousness, trauma, vomiting, chest pain, or changes in urinary or bowel habits. He had experienced a similar episode of rhabdomyolysis requiring hemodialysis in the past. He has a history of smoking, alcohol, and drug use and is currently not on any prescription medication.

Upon arrival in the emergency room, his vital signs were temperature 97.1°F, pulse 133/minute, blood pressure 135/89 mmHg, respiratory rate 23/ minute, and oxygen saturation of 95% on room air. The initial physical examination revealed an anxious patient, in moderate distress due to pain. He was alert and oriented, with rapid speech. His pupils were about 5mm, round and reactive, and his mucous membranes were dry. Movement was limited by pain in all four extremities. His urine was dark red in color. Initial laboratory investigations are shown in Table 1. The most significant finding was a total creatine phosphokinase of 118,104 U/L. Liver enzymes were also significantly elevated. The serum and urine myoglobin were not measured. The urine drug screening was negative, acetaminophen level was <2.0mcg/mL, acetylsalicylate <3.0mg/dL, and ethanol level <3.0mg/dL.

**Table 1:** Initial laboratory investigations ALT - Alanine Aminotransferase, AST - Aspartate Aminotransferase, BUN - Blood Urea Nitrogen, CK - Creatine Kinase, Cr - Creatinine, Hgb - Hemoglobin, Hct - Hematocrit, RBC - Red Blood Cell, WBC - White Blood Cell

Laboratory parameter	Reference value/units	Patient’s result
Biochemistry		
Sodium	136-145 mmol/L	140
Potassium	3.5-5.1 mmol/L	3.5
Bicarbonate	20 – 31 mmol/L	9.4
pH	7.35 – 7.45	6.92
BU	7-18 mg/dL	63
Cr	0.7-1.30 mg/dL	4.58
AST	<34 U/L	1399
ALT	10-49 U/L	240
Alkaline Phosphatase	46 - 116 U/L	69
Total CK	46-171 U/L	118108
Lactic acid	0.50-2.20 mmol/L	1.2
Serum osmolality	mOsm/Kg	294
Complete blood count		
WBC	4.0 - 11.2 K/UL	20.9
Hgb	13.7 - 17.5 g/dl	15.1
Hct	40.0 - 51.0 %	45.6
Platelet Count	150 - 400 k/ul	201
Urinalysis		
Urine pH	4.5-8	5.5
Urine Specific Gravity	1.005 - 1.030	1.021
Urine Blood	Negative	Small
Urine Casts	0-2 / LPF	0-2
Urine RBC	0-2 / HPF	0-2
Urine WBC	0-5 / HPF	0-5
Urine Osmolality	250 - 900 MOSM/KG	352

**Figure 1:**
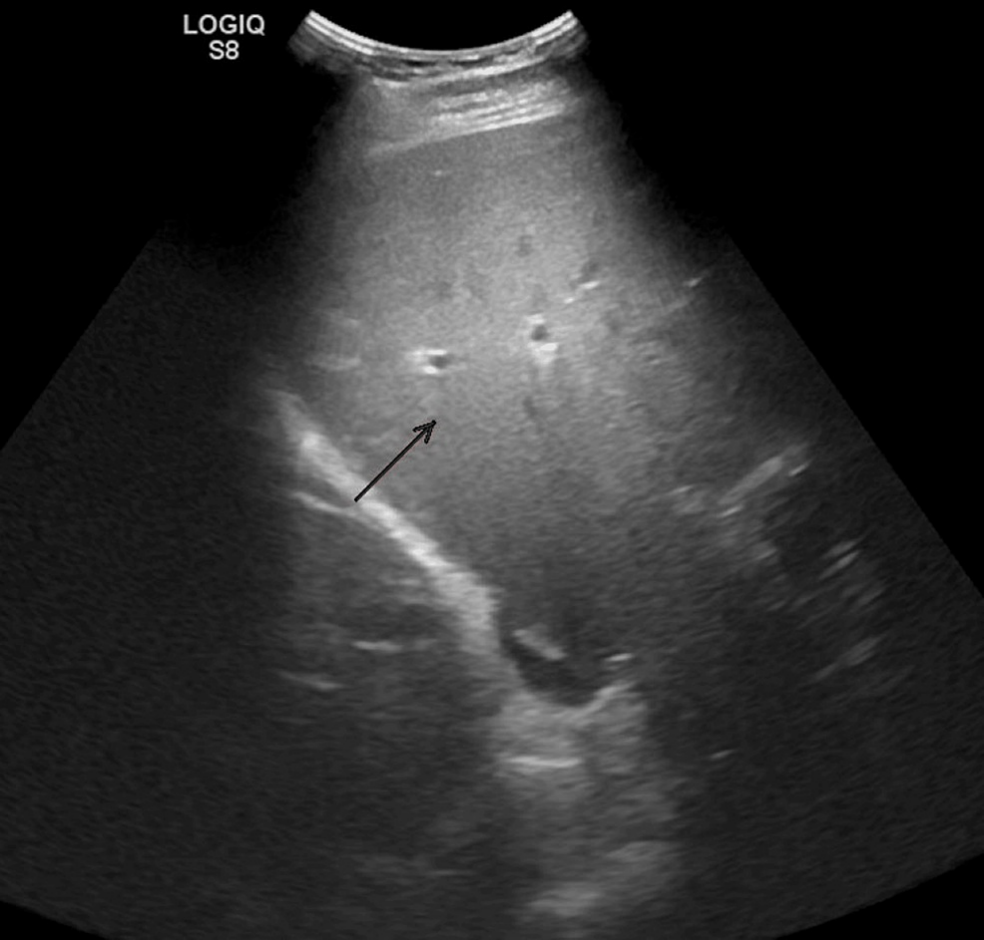
Ultrasound of the liver showing a heterogenous liver parenchyma with no intrahepatic or extrahepatic biliary ductal dilatation.

**Figure 2:**
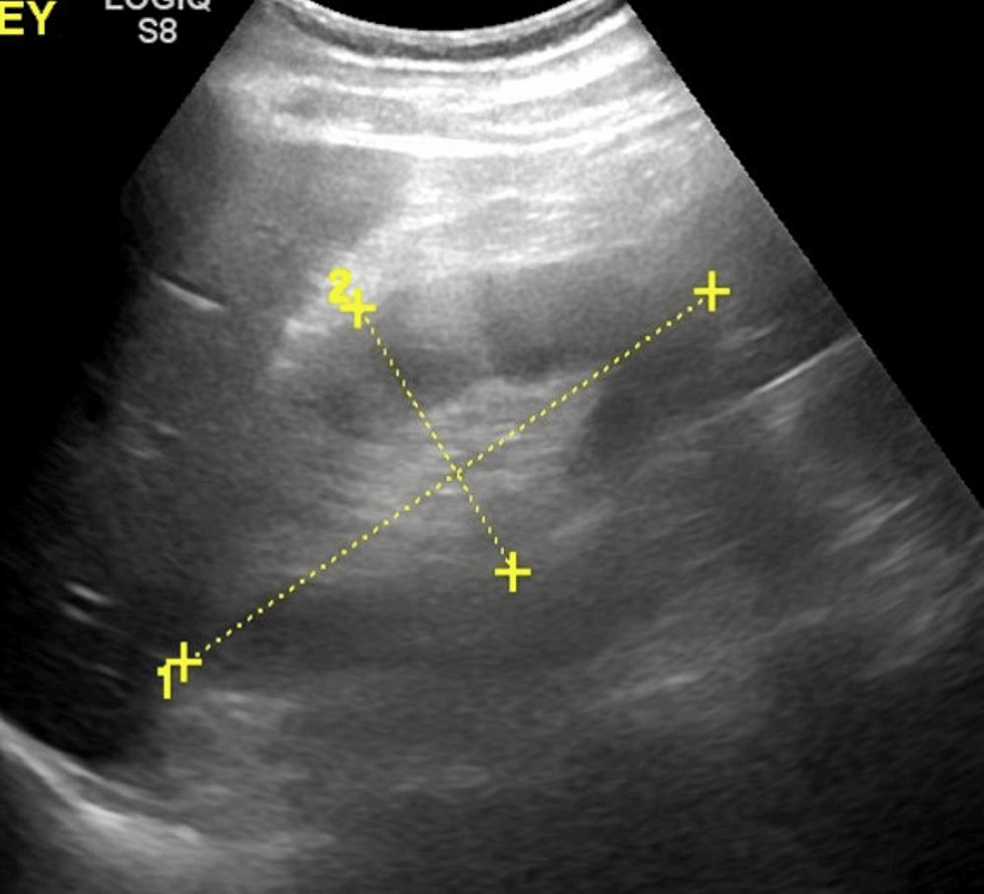
Ultrasound of the right kidney showing a normal kidney measuring approximately 12.6 x 5.9 x 5.3 cm. There were no cysts, solid mass lesions, hydronephrosis, or calculus, and cortical central differentiation was maintained in both kidneys.

**Figure 3:**
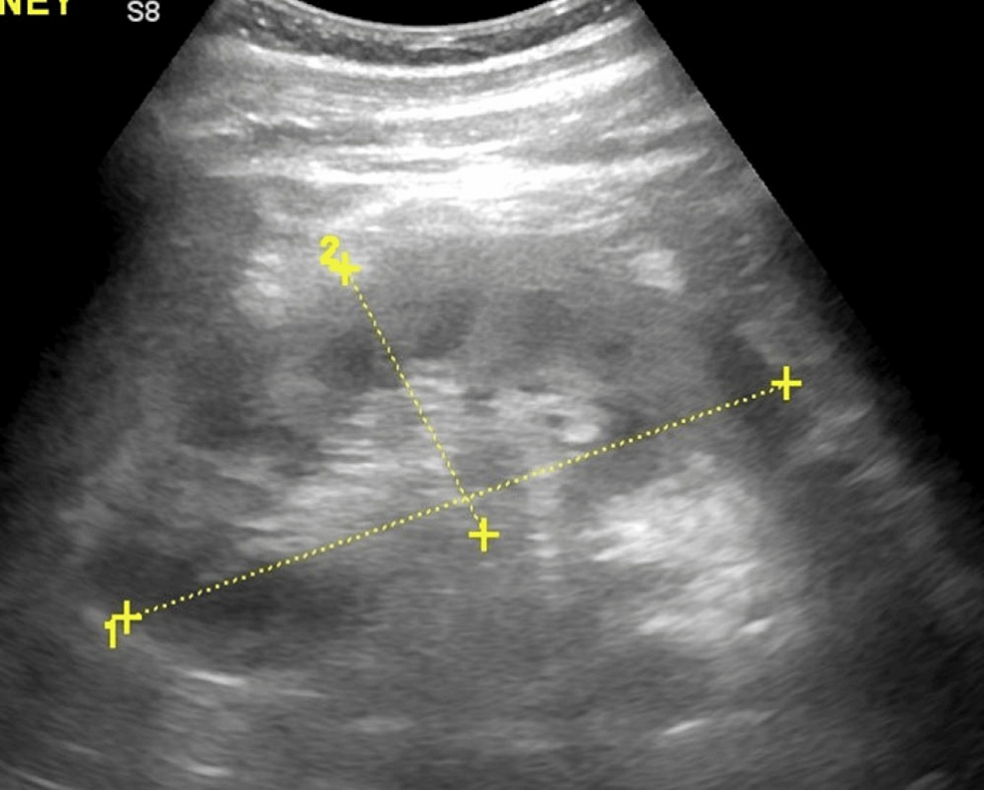
Ultrasound of the left kidney showing a normal kidney measuring approximately 12.4 x 5.3 x 5.7 cm. There were no cysts, solid mass lesions, hydronephrosis, or calculus, and cortical central differentiation was maintained in both kidneys.

An abdominal ultrasound showed a heterogeneous liver parenchyma. There was no intrahepatic or extrahepatic biliary ductal dilatation. The right kidney measured approximately 12.6 x 5.9 x 5.3 cm in length and the left kidney measured approximately 12.4 x 5.3 x 5.7 cm in length; neither kidney demonstrated a cyst, solid mass lesion, hydronephrosis or calculus, and cortical central differentiation was maintained in both kidneys. See Figures 1-3 for the liver, right, and left kidneys, respectively.

The patient was diagnosed with MDMA-induced severe rhabdomyolysis with AKI associated with severe metabolic acidosis. He was initially admitted to the ICU and a Foley catheter was inserted for accurate recording of input and output. He was started on aggressive intravenous fluids, as well as sodium bicarbonate drip. On day 2, the metabolic acidosis and oliguria persisted, the creatinine and creatine phosphokinase were trending up, and as a result, he was started on hemodialysis. Table 2 showed a trend of some laboratory findings and urine output during the course of admission.

**Table 2:** Trend of selected parameters ALT - Alanine Aminotransferase, AST - Aspartate Aminotransferase, BUN - Blood Urea Nitrogen, Cr - Creatinine, K - Potassium, Phos - Phosphate, Total CPK - Total Creatine Kinase, UO - Urine output

Parameter	Reference value/units	Day 1	Day 2	Day 3	Day 4	Day 5	Day 6	Day 7	Day 8	Day 9	Day 10	Day 11	Day 12	Day 13	Day 14
Cr	0.7-1.3 mg/dL	4.58	5.23	7.26	7.60	9.51	8.16	10.06	8.23	9.54	10.14	9.9	8.2	6.4	5.4
BUN	7-18 mg/dL	63	58	58	48	57	38	42	31	33	35	34	31	26	23
K	3.5-5.1 mmol/L	3.5	3.9	3.5	3.5	3.8	3.6	3.8	3.9	3.9	3.5	3.7	3.5	3.7	4.2
Phosphorus	2.4-5.1 mg/dL	-	5.5	4.9	3.9	3.7	2.5	3.0	3.1	3.8	4.4	5.5	6.2	5.5	5.5
AST	<34 U/L	1,399	1,976	1,384	872	526	310	185	123	87	59	45	-	-	-
ALT	10-49 U/L	240	414	430	370	290	232	189	149	127	101	91	-	-	-
Total CK	46-171 U/L	118,108	158,954	88,781	47,696	19,145	9,695	5,670	2,931	1,817	1,172	867	-	-	-
UO	ml/day	-	115.0	195.0	165.0	-	-	150.0	1,000.0	4,150.0	2,980.0	3,900	3,800	3,700	-

The patient was downgraded from the ICU on day 4. Nine days after hospitalization, he started having significant diuresis. A few days later, the creatinine levels started trending down and urine output stabilized. Hemodialysis was discontinued on day 8 and the patient was discharged after 14 days in hospital with a creatinine level of 5.4 mg/dL. The patient followed up in the outpatient nephrology clinic one week later, and the Cr was 1.9 showing progressive improvement. The patient was strongly advised to abstain from the use of illegal drugs.

## Discussion

Ecstasy or MDMA is a commonly abused drug, and its use is very high in the adolescent population, with a study in US college students conducted in 1988 revealing that 39% of students admitted to using ecstasy at least once in the past year [3]. According to Yang et al. in 2023 [4], about 0.9% of individuals use recreational ecstasy/MDMA. The risk of death from ecstasy in first-time users has been estimated to be between one in 2000 and one in 50,000 [5]. Though it has all the negative side effects, ecstasy was recently granted breakthrough therapy designation for the treatment of posttraumatic stress disorder by the US Food and Drug Administration in 2017 [4].

Ecstasy is an amphetamine derivative, and it causes the release of neuroactive compounds such as serotonin (5-hydroxytryptamine), dopamine, and norepinephrine into the central nervous system. It also inhibits the uptake of these neurotransmitters, especially serotonin, after their release from neurons, through its interaction with the membrane transporters involved in neurotransmitter reuptake and vesicular storage systems [6,7]. The result is an acute increase in the levels of these neurotransmitters at the synapse which are then responsible for the many effects that are induced by ecstasy use. Accumulation of serotonin can cause serotonin syndrome, which can affect thermoregulation and autonomic dysfunction and cause changes in a person’s mental condition, leading to autonomic hyperactivity (high blood pressure and heart rate, mydriasis) and neuromuscular abnormalities [2]. Using MDMA with medications that increase serotonin levels like selective serotonin reuptake inhibitors puts the patient at even higher risk for these side effects [8]. Ecstasy has also been documented as causing an increase in serum levels of prolactin, arginine vasopressin (AVP or ADH), cortisol, oxytocin, and adrenocorticotropic hormone [6].

The doses for typical recreational MDMA range from 1 to 2 mg/kg. MDMA has a half-life of 7.6 hours, but it begins to take effect after 30-60 min, peaking at 90 min with its effect generally lasting between four and six hours [6]. MDMA metabolizes in two pathways. The first pathway involves the conjugation with glycine, after undergoing N-dealkylation, deamination, and oxidation to become 4-hydroxy-3-methoxyamphetamine (HMA). The second pathway consists of the cytochrome P450 system isoenzyme CYP2D6 which is responsible for converting MDMA to its principal metabolite, 4-hydroxy-3-methoxymethamphe-tamine (HMMA), a potent inducer of ADH secretion, through O-demethylation and catechol-O-methyltransferase (COMT). The second pathway shows genetic variance and those who show slow metabolism are at higher risk for acute toxicity [3,6,7]. Concurrently taking medications that inhibit the cytochrome CYP2D6 isoenzyme like cimetidine, ritonavir, and antifungal medications will increase the risk of ecstasy toxicity [8].

The metabolism of MDMA is nonlinear with a slight increase in dose causing a large increase in the amount of MDMA in the blood. MDMA and its metabolites are cleared by the kidneys [2]. Due to personal pharmacokinetics, the concurrent use of MDMA with other medications, and impaired renal function, people taking the same dose of MDMA may experience side effects to differing degrees compared to others. MDMA can affect multiple organs, causing central nervous system, cardiac, muscular, renal, and hepatic dysfunction [9]. Our patient had a serious complication from MDA use probably from his personal pharmacokinetics.

Rhabdomyolysis is the disruption of skeletal muscle in which intracellular contents such as potassium, phosphorus, calcium, and myoglobin are released into the circulation. Elevated blood creatine phosphokinase (CK) is often used to screen patients for rhabdomyolysis [10]. In rhabdomyolysis, serum creatine kinase (CK) is elevated (>5× the upper limit of normal or >1,000 U/L) along with elevated serum myoglobin, lactate dehydrogenase potassium, creatinine, aspartate aminotransferase, alanine aminotransferase, and urine myoglobin [11]. The increase in serotonin levels caused by MDMA use not only directly triggers rhabdomyolysis but also causes hyperpyrexia, muscle stiffness, and hyperreflexia. This can also lead to rhabdomyolysis independent of environmental elements like hyperactivity, dehydration, or warm ambiance. Levels of creatinine phosphokinase can reach over 100,000 U/L, like in our patient, with a maximum reported level being 555,000 U/L after MDMA use [12].

As was the case in our patient, the most significant reason for AKI after MDMA use is acute tubular necrosis caused by non-traumatic rhabdomyolysis injury. Some studies show AKI is as high as 67% of rhabdomyolysis. AKI results from the effect of the heme-containing compound, myoglobin, causing renal tubular obstruction, direct renal toxicity, renal ischemia, and decreased glomerular permeability [13]. Increased reactive oxygen species with MDMA metabolism and toxic oxidation and glutathione depletion may be responsible for direct tissue damage not only to the brain, liver, and heart but also to the kidneys (causing AKI) and muscles causing rhabdomyolysis [2,14]. Volume depletion also increases the nephrotoxic effect of rhabdomyolysis.

Treatment of MDMA-induced rhabdomyolysis and AKI requires hydration, and monitoring of temperature, fluid, electrolytes, and renal function including intake and output [6,12]. Dialytic treatment is necessary to correct hydroelectrolytic imbalance and renal function alterations. Initiating hemodialysis early like in the case of our patient does not only avoid life-threatening complications but it is a pathogenetic treatment because it removes a great amount of myoglobin from the plasma [15].

## Conclusions

MDMA or ecstasy is an illegal drug that is commonly abused by adolescents and young adults. In addition to the euphoria and hallucinations, MDMA can have very serious side effects including rhabdomyolysis, which can progress to acute renal failure. Its action on serotonin neurotransmitters and cytochrome P450 isoenzymes can result in serious side effects when taken with some classes of medications. In susceptible patients, these side effects occur whenever the patient is exposed to the drug. Physicians have an obligation to recognize these deadly side effects of MDMA and start immediate and appropriate treatment. It is therefore very crucial to educate individuals about the dangerous effects of illicit drugs and discourage their use.
